# What Open-Lung Biopsy Teaches Us about ARDS in COVID-19 Patients: Mechanisms, Pathology, and Therapeutic Implications

**DOI:** 10.1155/2020/2909673

**Published:** 2020-12-09

**Authors:** Yassamine Abourida, Houssam Rebahi, Hajar Chichou, Hicham Fenane, Yassine Msougar, Anas Fakhri, Fatima Ezzahra Hazmiri, Ayman Ismail, Hanane Rais, Nabila Soraa, Mohammed Abdenasser Samkaoui

**Affiliations:** ^1^Department of Anesthesia and Intensive Care Medicine, Faculty of Medicine and Pharmacy of Marrakech, Cadi Ayyad University of Marrakech, Morocco; ^2^Laboratory of Childhood, Health & Development, Cadi Ayyad University of Marrakech, Morocco; ^3^Department of Thoracic Surgery, Mohammed VI University Hospital of Marrakech, Morocco; ^4^Laboratory of Histology and Embryology, Department of Preclinical Science, Faculty of Medicine and Pharmacy of Marrakech, Cadi Ayyad University of Marrakech, Morocco; ^5^Department of Pathology, Mohammed VI University Hospital of Marrakech, Morocco; ^6^Department of Microbiology, Mohammed VI University Hospital of Marrakech, Morocco

## Abstract

Difficulties have risen while managing Acute Respiratory Distress Syndrome (ARDS) caused by COVID-19, although it meets the Berlin definition. Severe hypoxemia with near-normal compliance was noted along with coagulopathy. Understanding the precise pathophysiology of this atypical ARDS will assist researchers and physicians in improving their therapeutic approach. Previous work is limited to postmortem studies, while our report addresses patients under protective lung mechanical ventilation. An open-lung minithoracotomy was performed in 3 patients who developed ARDS related to COVID-19 and were admitted to the intensive care unit to carry out a pathological and microbiological analysis on lung tissue biopsy. Diffused alveolar damage with hyaline membranes was found, as well as plurifocal fibrin microthrombi and vascular congestion in all patients' specimens. Microbiological cultures were negative, whereas qualitative Reversed Transcriptase Polymerase Chain Reaction (RT-PCR) detected SARS-CoV-2 in the pulmonary parenchyma and pleural fluid in two patients. COVID-19 causes progressive ARDS with onset of severe hypoxemia, underlying a dual mechanism: shunt effect through diffused alveolar damage and dead space effect through thrombotic injuries in microvascular beds. It seems reasonable to manage this ventilation-perfusion ratio mismatch using a high dose of anticoagulant combined with glucocorticoids.

## 1. Introduction

In the early months of 2020, the world witnessed an outbreak of the severe respiratory syndrome coronavirus (SARS-Cov-2), which caused a tremendous flood of coronavirus-related pneumonia. In most cases, coronavirus disease 2019 (COVID-19) is rapidly resolved, whereas 26% require intensive care unit admission [1].

Clinical manifestations of COVID-19 vary from mild pneumonia to progressive Acute Respiratory Distress Syndrome (ARDS). Its singular features are severe hypoxemia often associated with near-normal respiratory system compliance at the beginning [2]. On these distinctive grounds, a crucial question rose: How does COVID-19 damage the lungs to cause a rapidly progressive onset of profound hypoxemia?

In 1967, Ashbaugh et al. assumed that diffuse alveolar damage (DAD) on lung histology is the pathological hallmark of ARDS [3]. However, recent shreds of evidence point out that DAD is only a phenotype of ARDS among others, but with a higher mortality [4]. COVID-19 is a systemic disease that affects multiple organs, including the lungs, pharynx, heart, liver, brain, and kidneys [5]. Very little is known about the “weaponry” of COVID-19; however, its main target seems to be the vascular endothelium. Initial reports documented clinically significant coagulopathy in critically ill patients [6, 7]. According to the Berlin definition [8], diagnosing *ARDS* does not take into account pathologic findings which leaves a considerable gap in categorizing COVID-19 ARDS (C-ARDS) and its peripheral vascular changes. On the other hand, previous work studied the coinfection rate between SARS-CoV-2 and other respiratory pathogens and focused only on nasopharyngeal swabs [9] but failed to address it in the lower respiratory tract.

Understanding the precise pathophysiology of C-ARDS will assist researchers and physicians in improving their therapeutic approach. Here, we conducted a descriptive study performing an open-lung biopsy (OLB); taking into account the benefit-to-risk ratio, in patients with C-ARDS. This study is aimed at determining C-ARDS pathological characteristics and coinfection with other pathogens in lung tissue.

## 2. Methods

### 2.1. Patient and Diagnosis

We selected patients with laboratory-confirmed SARS-CoV-2 infection—admitted to the COVID-19 ICU of the University Hospital from the 25^th^ of April to 25^th^ June 2020—who later developed ARDS that met the Berlin definition [8] and were put under mechanical ventilation. Initial chest computed tomography (CT) scans revealed bilateral diffused ground-glass opacities in different percentages. Informed written consent from the next of kin was obtained. The ethics committees of the University Hospital approved this research respecting the regulations of the Helsinki declaration (ID UCA020-041).

### 2.2. Surgical Technique

**Figure 1:**
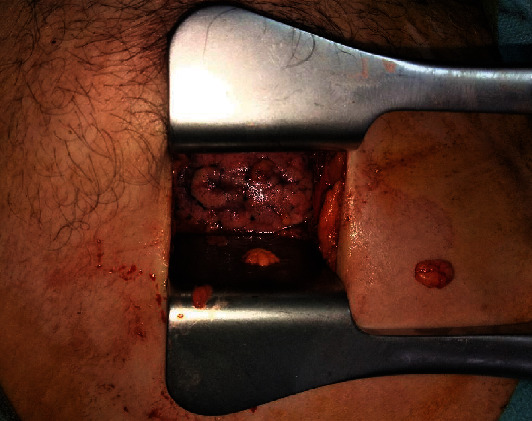
Rib spreading during minithoracotomy.

**Figure 2:**
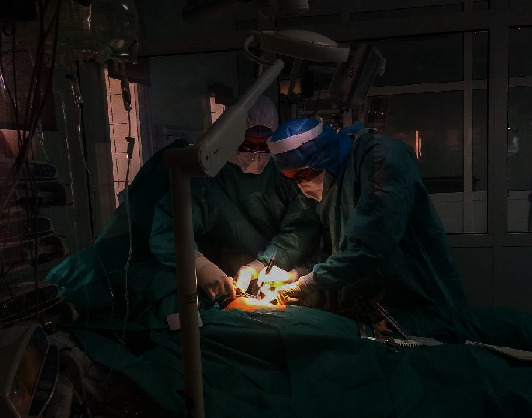
Surgical team performing open-lung biopsy while wearing appropriate personal protective equipment.

An open-lung minithoracotomy with rib spreading was performed using the wedge resection technique from the anterolateral segment with a stapler (Figure 1). The anterior end of the incision was placed 3 to 4 cm lateral to the middle line of the breastbone. Pleural space was entered above the fifth rib. A chest tube was placed through another incision, and the muscle layers were loosely closed with a running absorbable number 0 suture. A lung tissue fragment was immediately soaked in 4% formalin solution, and the other fragments along with pleural effusion fluid were put in a culture environment. The surgical team wore level 3 personal protective equipment during the invasive procedure (Figure 2).

### 2.3. Specimen's Analysis

Biopsy lung tissue was analyzed with hematoxylin-eosin and periodic acid-Schiff for detecting bacterial and fungal infection and also Masson trichrome staining to identify pulmonary interstitial fibrosis. The slides have been digitized using a Leica SCN400 Slide Scanner, and then images of tissue sections were captured. Pleural effusion fluid and biopsy lung tissue were tested for SARS-CoV-2 by RT-PCR and a panel of non-SARS-CoV-2 respiratory viral pathogens (adenovirus, coronavirus metapneumovirus, enterovirus, rhinovirus, MERS-CoV, parainfluenza virus, syncytial respiratory virus, and flu viruses A and B), along with standard bacterial and fungal respiratory cultures.

## 3. Results

### 3.1. Clinical Features

An open-lung biopsy in 3 patients with C-ARDS was carried out. All of them were male and tested positive for SARS-CoV-2 by nasopharyngeal swab at the time of hospital admission. The median age was 65 years (range, 57-72 years). The median duration from symptoms to admission is 10 days (range, 7-13days), and the median duration from admission to death was 9.6 days (range, 5-15 days). Initial symptoms in 3 patients were mainly fever, dry cough, and shortness of breath, whereas Case 3 reported anosmia.

Hypertension was found in all patients as preexisting comorbidity, Case 1 had hyperthyroidism and benign hypertrophy of the prostate, and Case 3 reported being a chronic smoker for 20 years with dyslipidemia (Table 1).

All patients were managed with the same national Moroccan protocol, which is hydroxychloroquine combined with azithromycin, zinc, vitamin C, and therapeutic dose of Low Molecular Weight Heparin (LMWH), in addition to acetylsalicylic acid. Two patients had A+ blood type and Case 1 had O+. The median duration of noninvasive ventilation management was 6.3 days (range, 3-10 days), and the median duration of mechanical ventilation was 3.3 days (range, 2-5 days). Open-lung biopsy was performed on the first day of endotracheal intubation in all patients.

### 3.2. Biological Results

D-dimer serum levels at the admission of Cases 1, 2, and 3 were 7.41 *μ*g/mL, 2.27*μ*g/mL, and 0.31 *μ*g/mL, respectively, whereas at their last day were elevated to 21.27*μ*g/mL, 22.95 *μ*g/mL, and 1.1 *μ*g/mL (Table 2). It is difficult to claim that none of the patients had thromboembolic events as no autopsy was performed.

### 3.3. Histological Results

**Figure 3:**
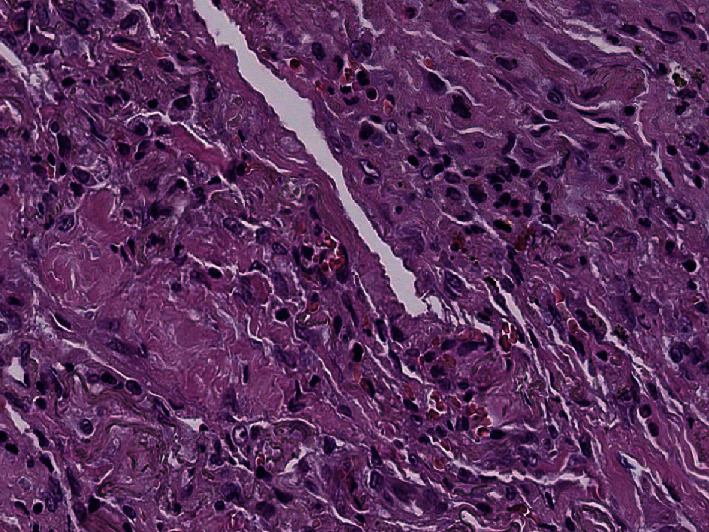
Thickening of the interalveolar walls (×10 magnification) (Case 2).

**Figure 4:**
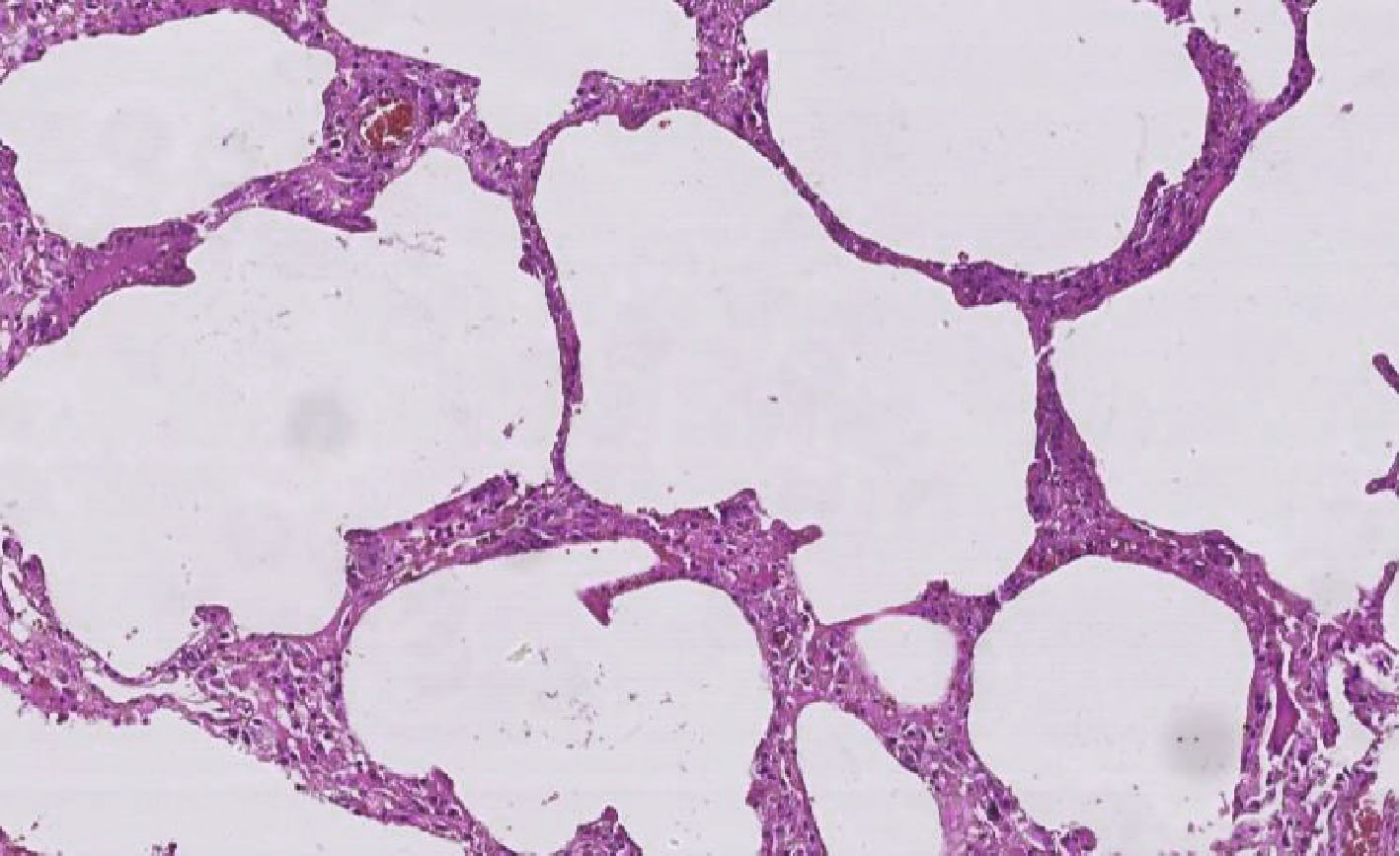
Enlarged alveolar airspace demonstrating emphysema (×20 magnification) (Case 3).

**Figure 5:**
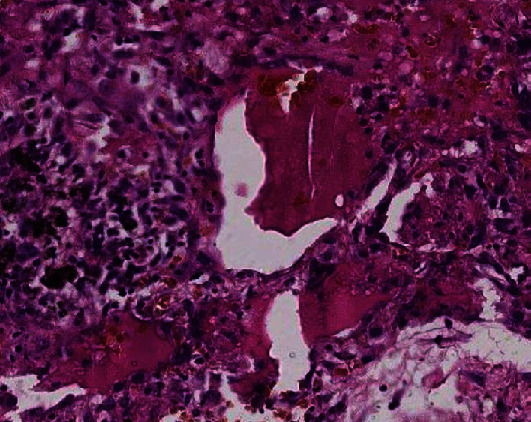
Protein exudates (×40 magnification) (Case 1).

**Figure 6:**
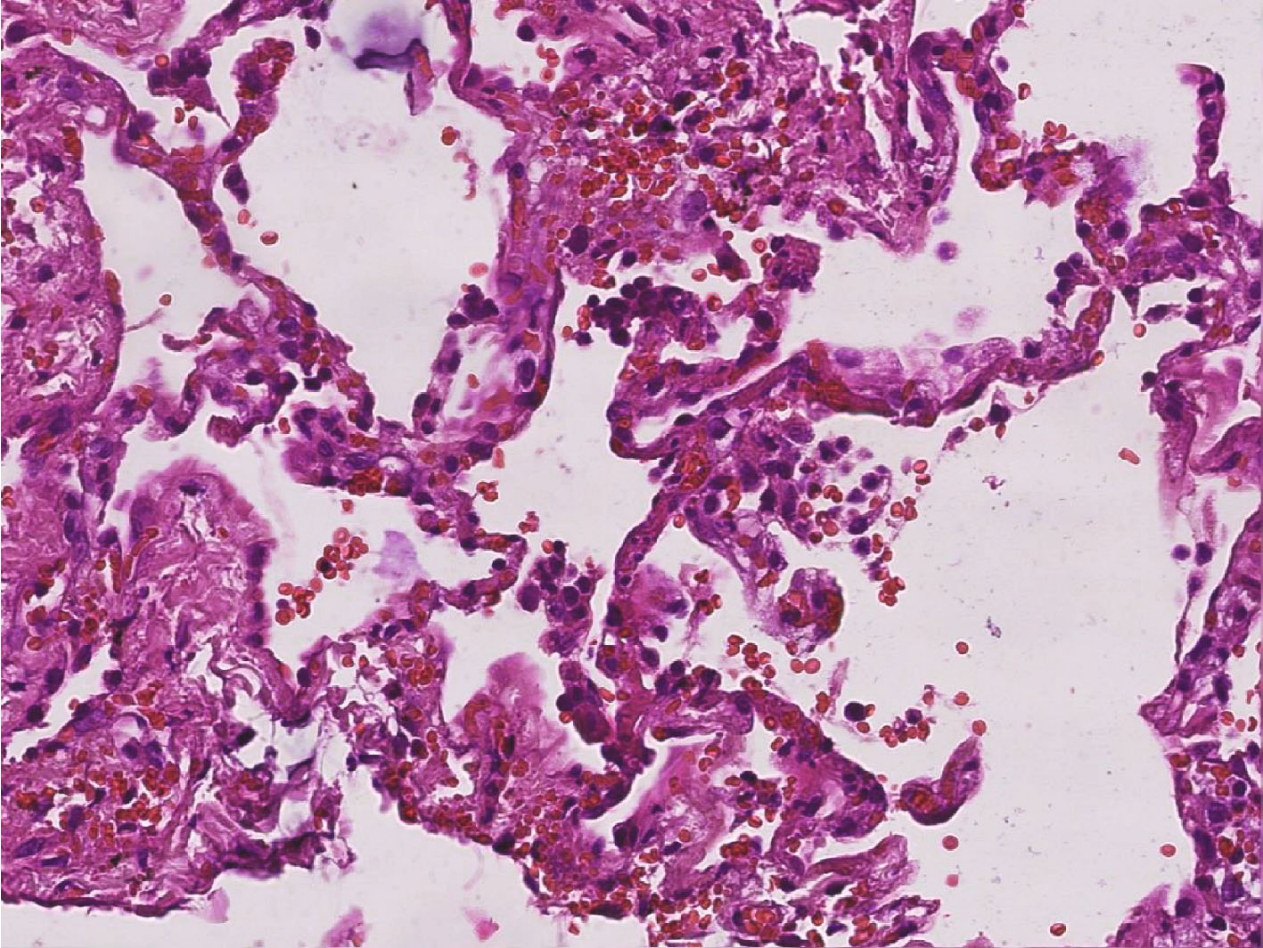
Type II pneumocytes displaying an atypical appearance with desquamation (×20 magnification) (Case 3).

**Figure 7:**
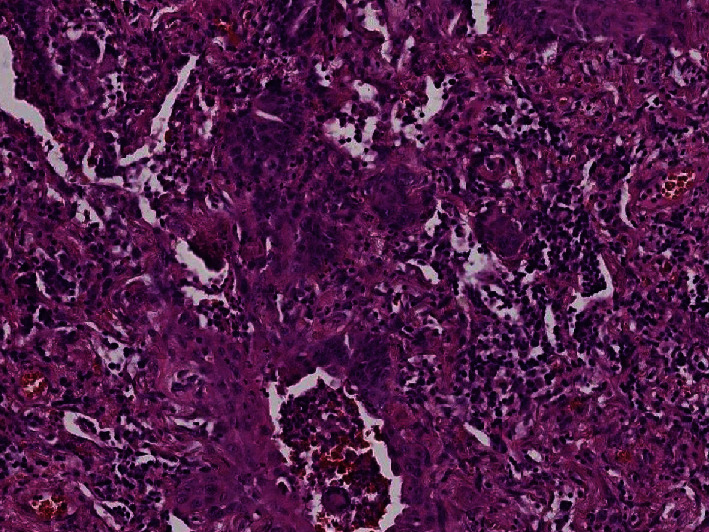
Fibroblastic multinucleated giant cells (×30 magnification) (Case 3).

**Figure 8:**
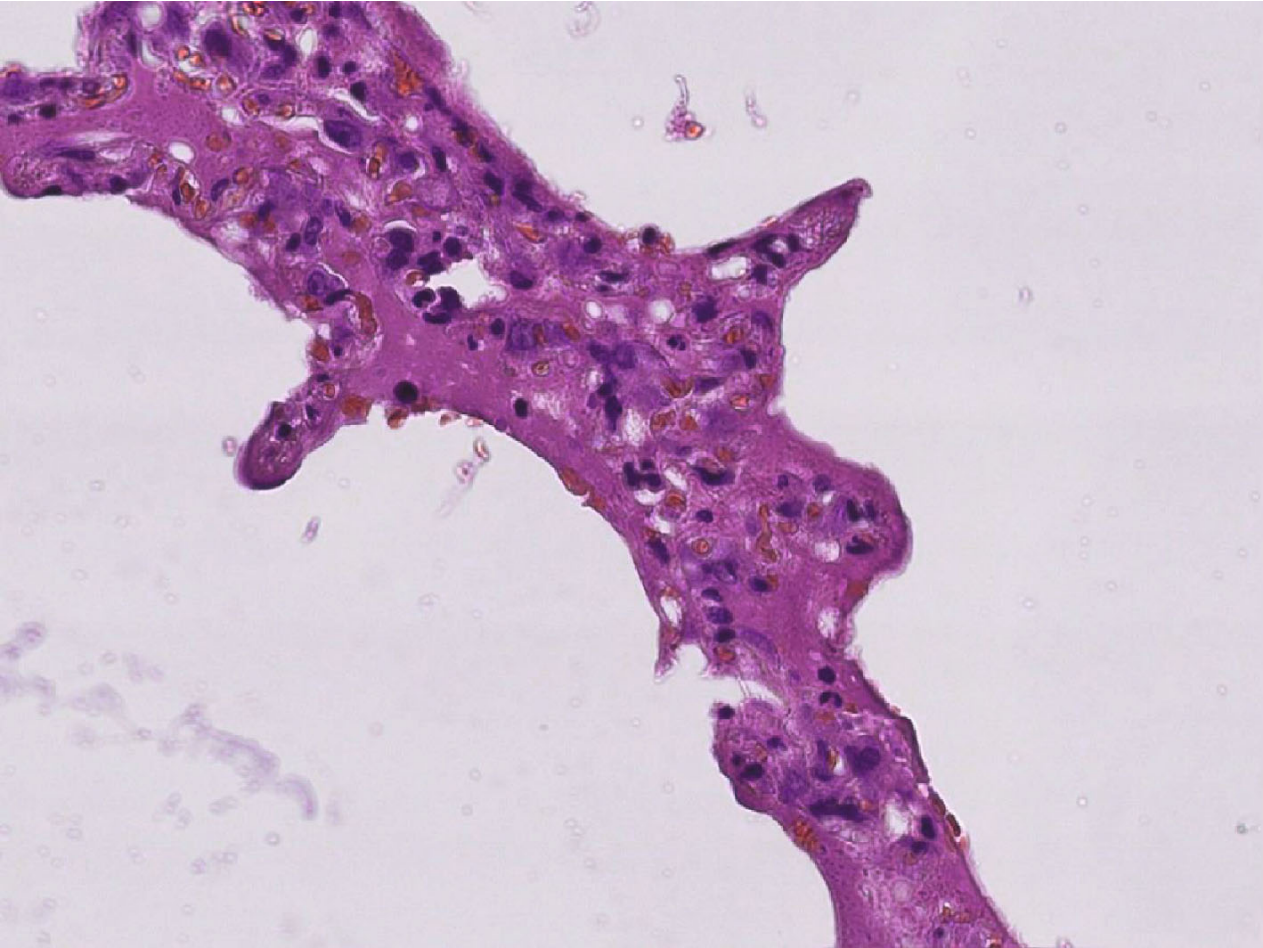
Hyaline membranes in the alveolar walls (×40 magnification) (Case 2).

**Figure 9:**
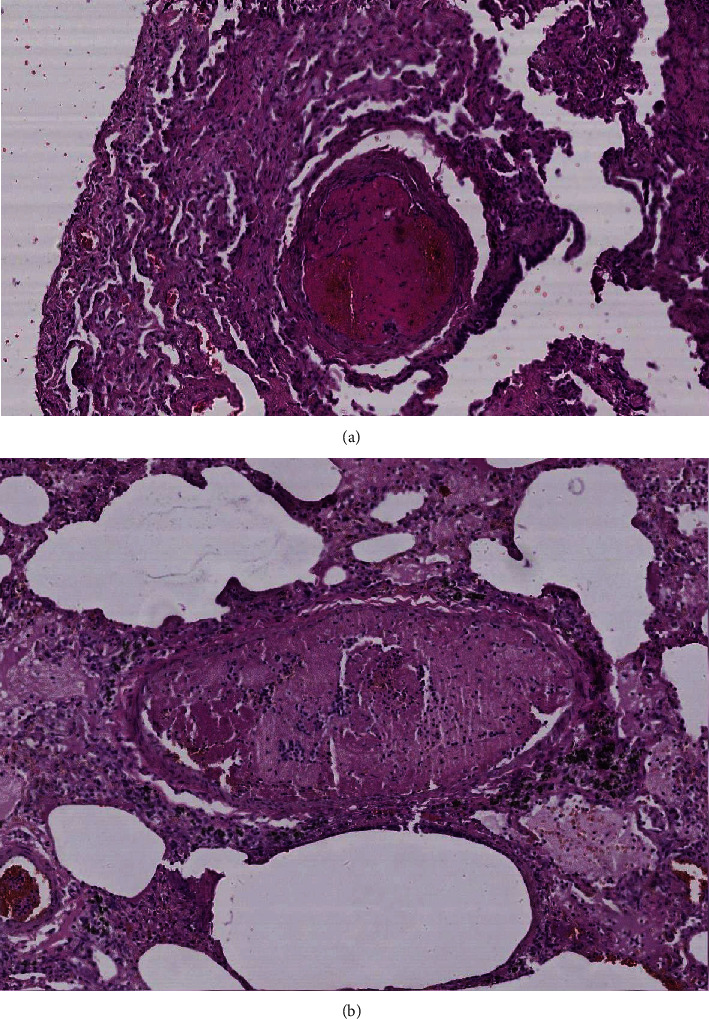
(a) Fibrinoid microthrombi (×10 magnification) (Case 2). (b) Fibrinoid microthrombi (×10 magnification) (Case 1).

**Figure 10:**
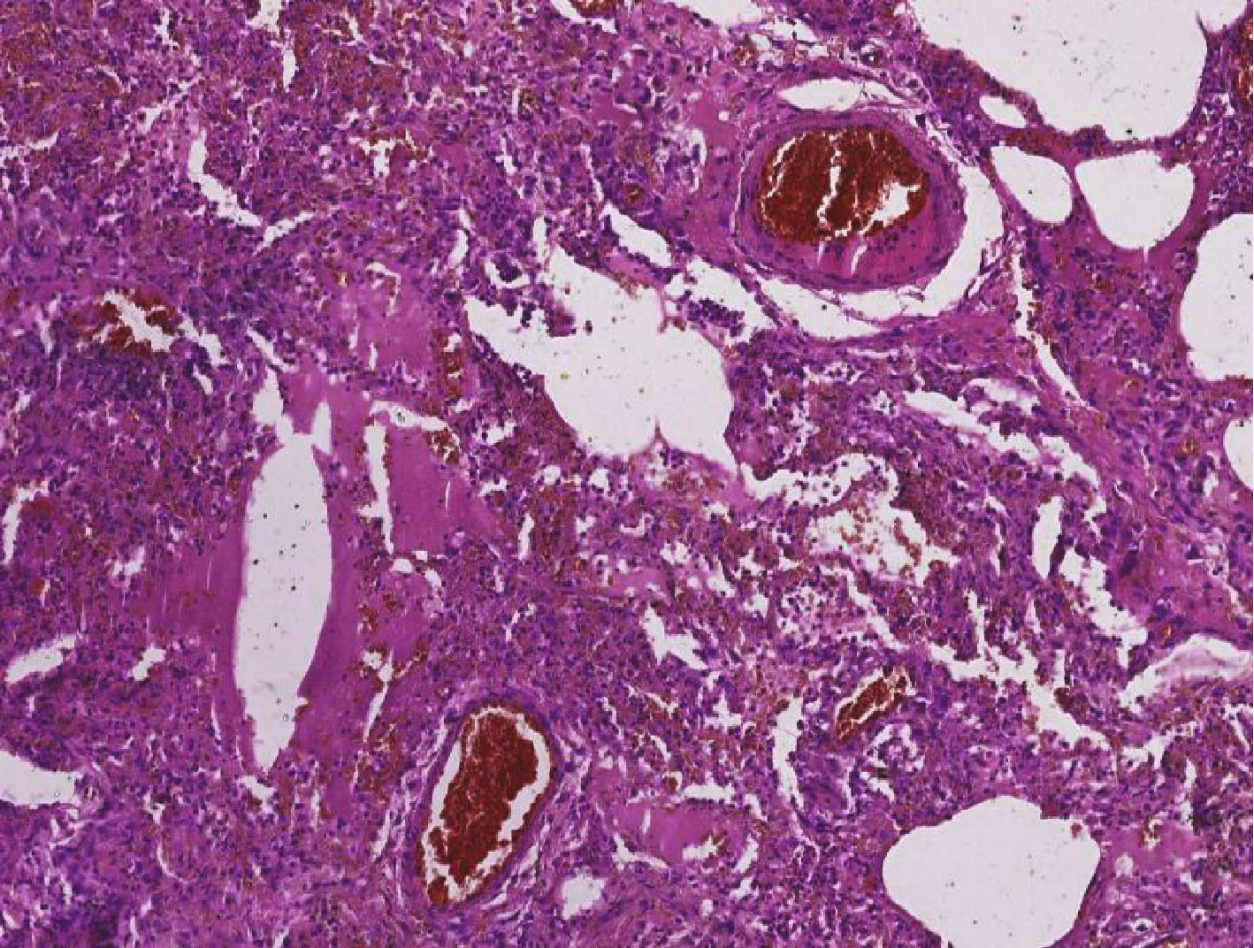
Vascular congestion (×10 magnification) (Case 2).

Histological examination showed diffuse alveolar damage with collapsed alveoli and intensified thickening of the intercellular septa in Cases 1 and 2 (Figure 3), whereas Case 3 exhibited enlarged airspaces, consistent with emphysema (Figure 4). The lumen was filled with proteinaceous and fibrin exudates (Figure 5). Type II pneumocytes were found hyperplasic with an atypical appearance, multinucleated with enlarged and prominent nuclei (Figure 6). There were significant focal points of pneumocyte desquamation, multinucleated giant cells (Figure 7), and hyaline membrane formation on the alveolar wall (Figure 8). Interstitial tissue displayed edema and widespread inflammatory infiltrates marked with lymphocytes mainly but also plasma cells, macrophages, and eosinophilic polynuclear cells. Prominent microthrombi (Figures 9(a) and 9(b)) and vascular congestion (Figure 10) were the major pathological finding in all cases. Also, a fibrin deposit was found in the vessel intima with a thickened vessel wall. Anthracosis deposit was also seen in Cases 1 and 2. No malignant tumor proliferation and no alveolar fibrosis were found in the 3 cases (Table 3). An additional file shows more of these results (see Additional file 1).

### 3.4. Microbiological Results

Bacterial and viral (other than SARS-CoV-2) culture returned negative. Qualitative RT-PCR detected SARS-CoV-2 in the pulmonary parenchyma and pleural fluid of Cases 2 and 3.

## 4. Discussion

Herein, an open-lung minithoracotomy was performed in 3 patients, who developed C-ARDS, in the first 24 hours of endotracheal intubation. Then, pathological, viral, and bacterial elements of lung tissues were examined. In all samples, the early phase of proliferative DAD was found associated with unexpected thrombosis of alveolar capillaries.

It seems plausible to hypothesize that, as a signature of ARDS, there were two intriguing mechanisms of hypoxemia and ventilation-to-perfusion ratio (V_A_/Q) mismatch caused by COVID-19. The first evident explanation is that thickening of alveoli walls and hyaline membranes is responsible for hypoxemia, but not solely [10]. The characteristics of type II pneumocytes suggest viral cytopathic-like changes, which could represent a direct attack on pulmonary parenchyma. Thus, collapsed alveoli arise in low ventilation, therefore a low V_A_/Q. Finding DAD in our patients' lung corroborates with previous postmortem pathological data of C-ARDS [11, 12] and also severe pneumonia due to influenza A/H1N1 [13]. It is important to mention that ventilation-induced lung injuries were significantly reduced since the biopsy was carried out on the first day of endotracheal intubation.

Second, the most striking result to emerge from the above data is the pattern of microthrombi formation in alveolar capillaries. New vessel growth through a mechanism of intussusceptive angiogenesis was reported recently [14]. Furthermore, pulmonary changes were observed and limited only to thrombotic microvascular injury with complement precipitation in lung septal microvasculature [15]. A large recent lung autopsy series points out the anticipated exudative phase of DAD with type II pneumocyte hyperplasia and interstitial edema, associated with congested capillaries and platelet-fibrin thrombi in small arterial vessels [16]. Hence, low perfusion of lungs results also in a high V_A_/Q, worsened by the loss of perfusion regulation. Both shunt effect and dead space contribute to the increase in minute ventilation and work of breathing and might simultaneously play a pivotal role in the heterogeneous pathogenesis of COVID-19 hypoxemia. Interestingly, it has now been suggested that respiratory failure caused by COVID-19 is not a typical ARDS [2].

In 2009, a novel swine-origin influenza A (H1N1) virus was responsible for a global pandemic alert [17]. Capelozzi et al. conducted an Open-Lung Biopsy in 5 patients with ARDS with confirmed H1N1, and the main pathological features found were necrotizing bronchiolitis, DAD, and alveolar hemorrhage, and it was hypothesized that DAD is a repercussion of bronchiolar obstruction [18], whereas in our patients, DAD is related to SARS-CoV-2 lesions. They also suggested that the bronchiolar epithelium was the primary target cells for swine-origin influenza A (H1N1), while SARS-CoV-2 damages mainly alveolar epithelial and endothelial cells.

It is noteworthy that fibrin thrombi were found in all patients as well as high levels of serum D-dimer. An increasing number of researchers highlighted coagulopathy abnormalities and thrombosis with severe COVID-19 patients [19, 20]. Moreover, this result has further strengthened the positive correlation between the elevated D-dimer level and the fatal prognosis of the disease course of COVID-19 [21]. In fact, a high incidence of thromboembolic events in patients with COVID-19 has been described in an autopsy series [22].

Most host responses in severe cases of COVID-19 reflect hyperinflammation and cytokine storm [23], which leads to intense recruitment and infiltration of monocytes, macrophages, and neutrophils in alveolar capillary beds, as in SARS-CoV-1 [24]. These infiltrates are known to produce uncontrolled proinflammatory chemokines and therefore intensify the vicious hyperinflammation cycle.

The presence of SARS-CoV-2 viral elements within the endothelial cells and the accumulation of inflammatory cells [25] may generate a prothrombotic state by activating endothelial coagulation factors. Thus, unrestrained and extensive immunothrombosis may increase the severity of microangiopathy [26], notably in older patients with cardiovascular comorbidities. A hypercoagulable state is allied with a relative hypofibrinolysis imbalance and results in fibrin deposits in the intra-alveolar space [27]. Severe hypoxemia resulting initially from the V_A_/Q mismatch might be the last straw to further amplify inflammation and increase blood viscosity [26], through activating platelets and plasma coagulation as well as through inhibiting the anticoagulant protein S [28].

In this study, bacterial and fungal cultures were negative, in contrast with the recent finding of bacterial abscesses in 4 patients among 38 who succumbed to COVID-19, with a single fungal abscess in one [16]. SARS-CoV-2 RT PCR was positive in two of our patients, whereas other respiratory pathogens were not present in lung tissue. Coinfection with other pathogens cannot be ruled out since it could be present in another pulmonary parenchyma that is not included in the biopsy fragment.

This research was carried out primarily to formulate adequate therapeutic strategies based on the uncovered pathogenesis of C-ARDS. Increasingly, it was pointed out that LMWH administration is tied to a better outcome in patients with severe COVID-19 that are showing an elevated level of D-dimers [29]. In addition to anticoagulation, heparin has anti-inflammatory properties that may be helpful to manage this disease but also provide endothelium protection [30].

There are several possible explanations for the inefficacy of LMWH administered in our patients. On the one hand, perhaps an asymptomatic pulmonary embolism was already present before the hospitalization of our hypercoagulable patients [31], considering their advanced age and their cardiovascular comorbidities but also the delay between the symptom onset and their hospitalization. On the other hand, it seems plausible that patients with C-ARDS require higher dose anticoagulation for preventing microvascular thrombosis and endothelial injury, in the absence of any medical contraindication. In this setting, randomized clinical trials studying the efficacy and safety of the full dose of LMWH or UFH (unfractionated heparin) are urgently needed and are being conducted worldwide (NCT04401293, NCT04409834, and NCT04362085), as no data is available at this time for this indication. Even though minimal alveolar hemorrhage was found in the biopsy, managing the plurifocal fibrin microthrombi outweighs the risk of bleeding, which seems to be consistent with the low incidence of hemorrhagic complications in patients with severe COVID-19 coagulopathy [29]. Thus, a pragmatic necessity to use an intermediate/therapeutic dose of anticoagulation in this setting imposes itself.

From another angle, these findings would seem to support the role of corticosteroid therapy. It is well known that glucocorticoids reduce endothelial leakage through decreasing capillary permeability and lower leukocyte migration to the inflammation site, while effectively stopping the inflammatory cascade. It is interesting to note that concerning C-ARDS, it was reported that methylprednisolone appears to have reduced the risk of death [32]; moreover, a high dose (1000 or 500 mg/day) and short term use of methylprednisolone provide a better prognosis of patients with C-ARDS [33]. Although the World Health Organization (WHO) does not recommend routine use of corticosteroids in patients with SARS-CoV-2 [34], it was found that severe COVID-19 patients who received methylprednisolone improved their oxygenation significantly with no negative impact on viral clearance [35]. On another note, early use of dexamethasone was proven to lower the duration of mechanical ventilation and improve survival [36]. A preliminary report of the RECOVERY trial indicated that the administration of 6 mg of dexamethasone reduced mortality by 35% in patients with COVID-19, receiving invasive mechanical ventilation [37].

The present study has not investigated the samples using immunohistochemical staining for additional insights, due to lack of materials. Despite the sample size, we believe our preliminary work could be a starting point to further provide concrete and substantial answers regarding the mechanism and pathogenesis of severe hypoxemia in C-ARDS and its management.

## 5. Conclusion

The evidence from this study allows us to not only hypothesize that SARS-CoV-2 directly attacks lung alveoli leading to diffuse alveolar damage and more particularly plurifocal fibrin microthrombi in the peripheral vasculature beds, but also elucidate the mechanism behind V_A_/Q mismatch in severe C-ARDS hypoxemia. Additionally, this paper points out the crucial benefit of anticoagulant therapy and corticosteroids in terms of dead space and shunt effect, respectively. Further investigation should be conducted in order to determine pertinently C-ARDS hypoxemia mechanisms and the proper ventilation and therapeutic management.

## Figures and Tables

**Table 1:** Clinical, radiological, and microbiological characteristics of patients and treatment received during ICU stay.

	Case 1	Case 2	Case 3
Age (years)	72	68	59
Gender	Male	Male	Male
Known comorbidities	Hypertension	Hypertension	Hypertension
	Hyperthyroidism		
	Benign hypertrophy of the prostate		Dyslipidemia
Symptoms	Fever 38.7°C	Fever 38.9°C	Fever 38.5°C
	Dry cough	Dry cough	Dry cough
	Shortness of breath	Shortness of breath	Anosmia
	Thoracic pain	Fatigue	
Symptom duration before admission (days)	13	7	10
Admission to death (days)	15	5	9
Physical examination in admission	RR = 33 cpm	RR = 36 cpm	RR = 34 cpm
	SpO_2_ = 73%	SpO_2_ = 84%	SpO_2_ = 83%
	HR = 95 bpm	HR = 95 cpm	HR = 88 cpm
	BP: 110/60 mmHg	BP = 120/60 mmHg	BP = 130/80 mmHg
	Blood sugar: 1.44 g/L	Blood sugar: 1.7 g/L	Blood sugar: 3.5 g/L
Thoracic CT scan	Ground glass	Ground glass > 75%	Ground glass > 80%
	Crazy paving > 70%		
Blood culture	Multiresistant	Sterile	Sterile
	Acinetobacter baumannii		
	Gram-positive bacteria sensitive to colistin (catheter related infection)		
Duration of ventilator management (days)	CPAP = 10	CPAP = 3	CPAP = 6
	IV = 5	IV = 2	IV = 3
Treatment	Hydroxychloroquine		
	Antibiotics		
	Therapeutic dose of LMWH		
	Acetylsalicylic acid		
	Methylprednisolone		
	Zinc		
	Vitamin C		
	Acetaminophen		
	Carbimazole		
	Tocilizumab
Blood type	O+	A+	A+
Microbiology (lung tissue and pleural fluid)	Negative	Negative	Negative
SARS-CoV-2 on pleural fluid	Negative	Positive	Positive
SARS-CoV-2 on lung biopsy	Negative	Positive	Positive

RR: respiratory rate; HR: heart rate; SpO_2_: pulsed oxygenation saturation; BP: blood pressure; CPAP: continuous positive airway pressure; IV: invasive ventilation.

**Table 2:** D-dimer level in patient's serum during ICU stay (*μ*g/mL).

	Day 1	Day 4	Day 5	Day 7	Day 9	Day 10	Day 12
Case 1	7.41	7.53	8.22	9.83	11.72	14.95	21.27
Case 2	2.27	7.25	22.95				
Case 3	0.31	0.37	0.91	1.1			

**Table 3:** Histological features of lung biopsy patients.

	Case 1	Case 2	Case 3
Alveoli	Variable size	Variable size	Enlarged +++
	Collapsed +++	Collapsed +++	Collapsed +
		Enlarged +	
Interalveolus wall	Thickened +++	Thickened +++	Thickened ++
			Dystrophic +
Type II pneumocyte	(i) Hyperplasic +++ (ii) Atypical (iii) Multinucleated (iv) Enlarged	(i) Hyperplasic +++ (ii) Atypical (iii) Multinucleated (iv) Enlarged	(i) Discontinuous (ii) Hyperplasic ++
Alveolar cavity:			
(i) hyaline membrane (ii) exudate (iii) alveolar hemorrhage	+++	+++	++
	+++	+++	++
	+	0	+
Interstitial tissue: (i) inflammatory infiltrate	(i) Diffused (ii) Minimal (iii) Lymphocyte +++ (iv) Eosinophilic polynuclear ++	(i) Diffused (ii) Minimal (iii) Lymphocyte +++ (iv) Eosinophilic polynuclear ++ (v) Neutrophil polynuclear ++ (vi) Multinucleated giant cells +	(i) Diffused (ii) Minimal (iii) Lymphocyte + (iv) Plasmocyte ++ (v) Macrophage + (vi) Fibroblast ++
Microthrombi	+ + +	+ + +	+ +
Vascular congestion	+ + +	+ + +	+ + +
Consolidation	45%	55%	60%
Alveolar fibrosis (*Masson trichrome staining*)	Negative	Negative	Negative
Coinfection (*periodic acid-Schiff staining*)	Negative	Negative	Negative

The intensity was appreciated independently by two investigators and estimated. “+”: focal; “++”: plurifocal; “+++”: diffuse.

## Data Availability

All data generated or analyzed during this study are included in this published article and its supplementary information files.

## Supplementary Materials

Supplementary Materials The supplementary material has been provided by the authors to present readers with further microscopic images related to their work.

## References

[1] Wang D., Hu B., Hu C. (2020). Clinical characteristics of 138 hospitalized patients with 2019 novel coronavirus–infected pneumonia in Wuhan, China. *JAMA*.

[2] Gattinoni L., Coppola S., Cressoni M., Busana M., Rossi S., Chiumello D. (2020). COVID-19 does not lead to a “typical” acute respiratory distress syndrome. *American Journal of Respiratory and Critical Care Medicine*.

[3] Ashbaugh D. G., Bigelow D. B., Levine B. E. (1967). Acute respiratory distress in adults. *The Lancet*.

[4] Thompson B. T., Guérin C., Esteban A. (2016). Should ARDS be renamed diffuse alveolar damage?. *Intensive Care Medicine*.

[5] Puelles V. G., Lütgehetmann M., Lindenmeyer M. T. (2020). Multiorgan and renal tropism of SARS-CoV-2. *New England Journal of Medicine*.

[6] Zhang Y., Xiao M., Zhang S. (2020). Coagulopathy and antiphospholipid antibodies in patients with Covid-19. *The New England Journal of Medicine*.

[7] Escher R., Breakey N., Lämmle B. (2020). Severe COVID-19 infection associated with endothelial activation. *Thrombosis Research*.

[8] The ARDS Definition Task Force (2012). Acute respiratory distress syndrome: the Berlin definition. *JAMA*.

[9] Kim D., Quinn J., Pinsky B., Shah N. H., Brown I. (2020). Rates of co-infection between SARS-CoV-2 and other respiratory pathogens. *Journal of the American Medical Association*.

[10] Wheeler A. P., Bernard G. R. (2007). Acute lung injury and the acute respiratory distress syndrome: a clinical review. *The Lancet*.

[11] Zhang H., Zhou P., Wei Y. (2020). Histopathologic changes and SARS-CoV-2 Immunostaining in the lung of a patient with COVID-19. *Annals of Internal Medicine*.

[12] Schaller T., Hirschbühl K., Burkhardt K. (2020). Postmortem examination of patients with COVID-19. *JAMA*.

[13] Harms P. W., Schmidt L. A., Smith L. B. (2010). Autopsy findings in eight patients with fatal H1N1 influenza. *American Journal of Clinical Pathology*.

[14] Ackermann M., Verleden S. E., Kuehnel M. (2020). Pulmonary vascular endothelialitis, thrombosis, and angiogenesis in Covid-19. *New England Journal of Medicine*.

[15] Magro C., Mulvey J. J., Berlin D. (2020). Complement associated microvascular injury and thrombosis in the pathogenesis of severe COVID-19 infection: a report of five cases. *Translational Research*.

[16] Carsana L., Sonzogni A., Nasr A. (2020). Pulmonary post-mortem findings in a series of COVID-19 cases from northern Italy: a two-centre descriptive study. *The Lancet Infectious Diseases*.

[17] WHO (2020). *World now at the start of 2009 influenza pandemic*.

[18] Capelozzi V. L., Parra E. R., Ximenes M., Bammann R. H., Barbas C. S. V., Duarte M. I. S. (2010). Pathological and ultrastructural analysis of surgical lung biopsies in patients with swine-origin influenza type A/H1N1 and acute respiratory failure. *Clinics*.

[19] Levi M., Thachil J., Iba T., Levy J. H. (2020). Coagulation abnormalities and thrombosis in patients with COVID-19. *The Lancet Haematology*.

[20] Klok F. A., Kruip M. J. H. A., van der Meer N. J. M. (2020). Incidence of thrombotic complications in critically ill ICU patients with COVID-19. *Thrombosis Research*.

[21] Li Y., Zhao K., Wei H. (2020). Dynamic relationship between D-dimer and COVID-19 severity. *British Journal of Haematology*.

[22] Wichmann D., Sperhake J.-P., Lütgehetmann M. (2020). Autopsy findings and venous thromboembolism in patients with COVID-19: a prospective cohort study. *Annals of Internal Medicine*.

[23] Mehta P., McAuley D. F., Brown M., Sanchez E., Tattersall R. S., Manson J. J. (2020). COVID-19: consider cytokine storm syndromes and immunosuppression. *The Lancet*.

[24] Channappanavar R., Fehr A. R., Vijay R. (2016). Dysregulated type I interferon and inflammatory monocyte-macrophage responses cause lethal pneumonia in SARS-CoV-infected mice. *Cell Host & Microbe*.

[25] Varga Z., Flammer A. J., Steiger P. (2020). Endothelial cell infection and endotheliitis in COVID-19. *The Lancet*.

[26] Henry B. M., Vikse J., Benoit S., Favaloro E. J., Lippi G. (2020). Hyperinflammation and derangement of renin-angiotensin-aldosterone system in COVID-19: a novel hypothesis for clinically suspected hypercoagulopathy and microvascular immunothrombosis. *Clinica Chimica Acta*.

[27] Whyte C. S., Morrow G. B., Mitchell J. L., Chowdary P., Mutch N. J. (2020). Fibrinolytic abnormalities in acute respiratory distress syndrome (ARDS) and versatility of thrombolytic drugs to treat COVID-19. *Journal of Thrombosis and Haemostasis*.

[28] Schulman S. (2020). Coronavirus disease 2019, prothrombotic factors, and venous thromboembolism. *Seminars in Thrombosis and Hemostasis*.

[29] Tang N., Bai H., Chen X., Gong J., Li D., Sun Z. (2020). Anticoagulant treatment is associated with decreased mortality in severe coronavirus disease 2019 patients with coagulopathy. *Journal of Thrombosis and Haemostasis*.

[30] Thachil J. (2020). The versatile heparin in COVID-19. *Journal of Thrombosis and Haemostasis*.

[31] Marietta M., Ageno W., Artoni A. (2020). COVID-19 and haemostasis: a position paper from Italian society on thrombosis and haemostasis, SISET. *Blood Transfusion*.

[32] Wu C., Chen X., Cai Y. (2020). Risk factors associated with acute respiratory distress syndrome and death in patients with coronavirus disease 2019 pneumonia in Wuhan, China. *JAMA Internal Medicine*.

[33] So C., Ro S., Murakami M., Imai R., Jinta T. (2020). High-dose, short-term corticosteroids for ARDS caused by COVID-19: a case series. *Respirology Case Reports*.

[34] WHO (2020). Clinical management of severe acute respiratory infection (SARI) when COVID-19 disease is suspected. https://www.who.int/docs/default-source/coronaviruse/clinical-management-of-novel-cov.pdf.

[35] Liu J., Zheng X., Huang Y., Shan H., Huang J. (2020). Successful use of methylprednisolone for treating severe COVID-19. *Journal of Allergy and Clinical Immunology*.

[36] Villar J., Ferrando C., Martínez D. (2020). Dexamethasone treatment for the acute respiratory distress syndrome: a multicentre, randomised controlled trial. *The Lancet Respiratory Medicine*.

[37] The RECOVERY Collaborative Group (2020). Dexamethasone in hospitalized patients with Covid-19 - preliminary report. *New England Journal of Medicine*.

