# Supplementary material for: What Open-Lung Biopsy Teaches Us about ARDS in COVID-19 Patients: Mechanisms, Pathology, and Therapeutic Implications
Source: Biomed Res Int. 2020 Dec 9;2020:2909673. doi: 10.1155/2020/2909673 (PMC7744583; doi:10.1155/2020/2909673)
Supplement: Supplementary Materials — The supplementary material has been provided by the authors to present readers with further microscopic images related to their work. [file 2909673.f1.docx]

**Supplementary Materials 1**

*Case 1 pathological findings:*


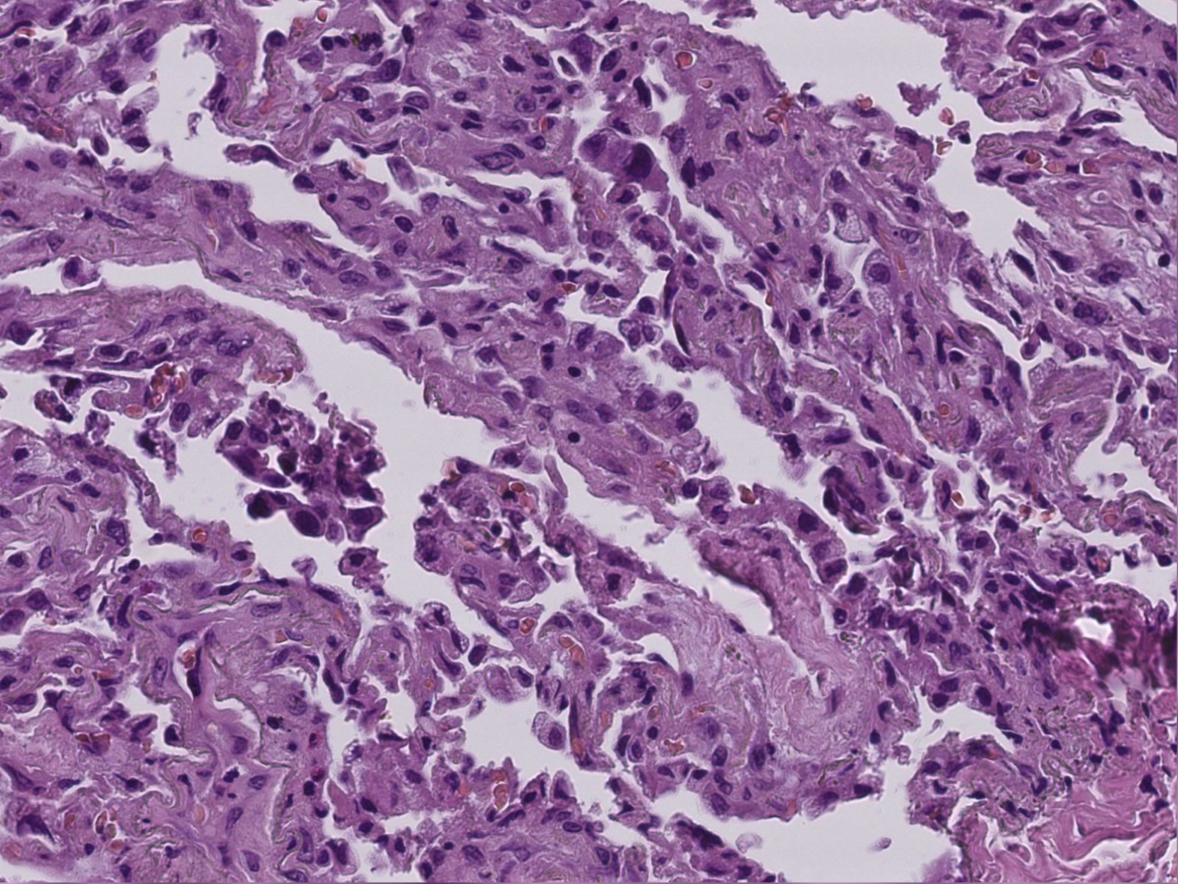


**Figure 1.** *Type II pneumocyte features.* (X20).

Desquamated hyperplasic pneumocytes filling the alveolar lumen with increased number of type II pneumocytes lining the alveolar wall.


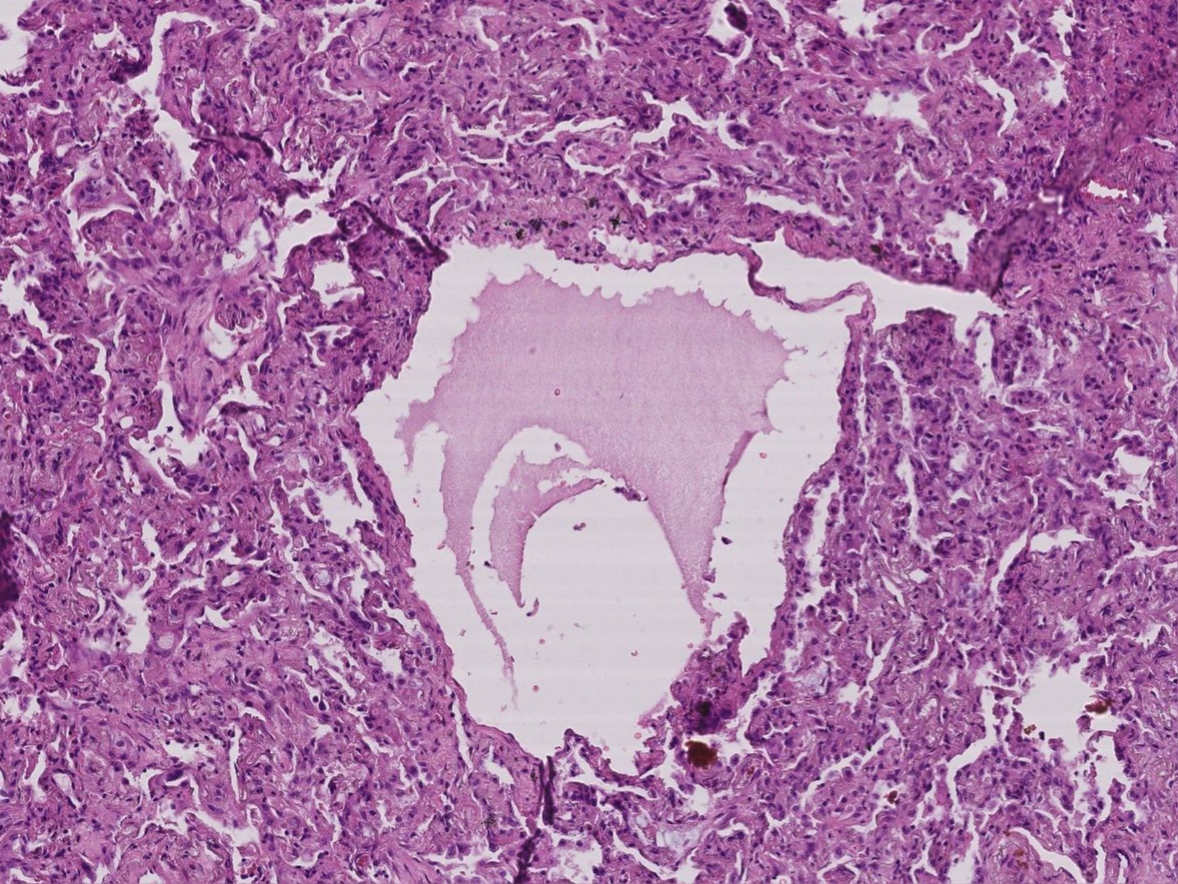


**Figure 2.** *Protein exudates* (x20 magnification).

Rich fluid in protein and cells leak through their alveolar walls.


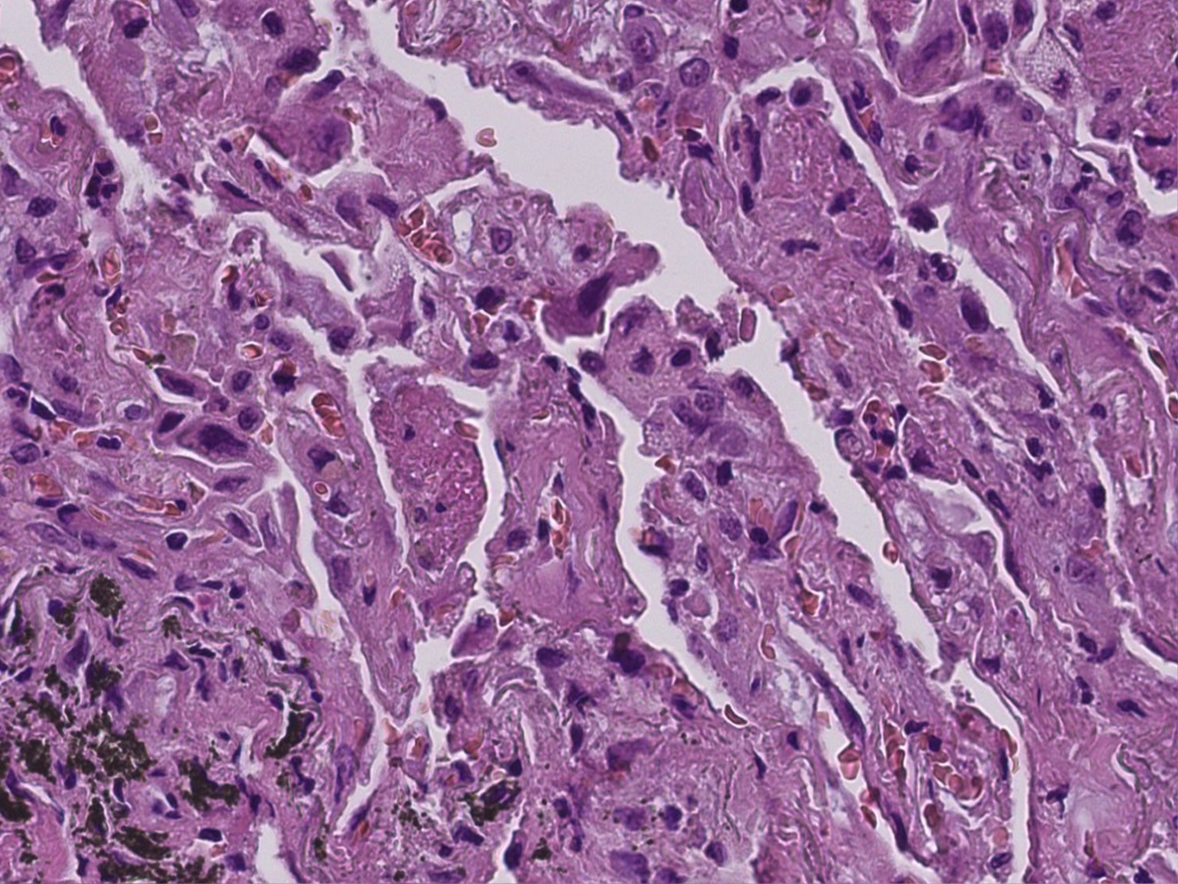


**Figure 3.** *Intra-alveolar fibrin deposit.* (x 20 magnification)

Fibrin within alveolar spaces.

**
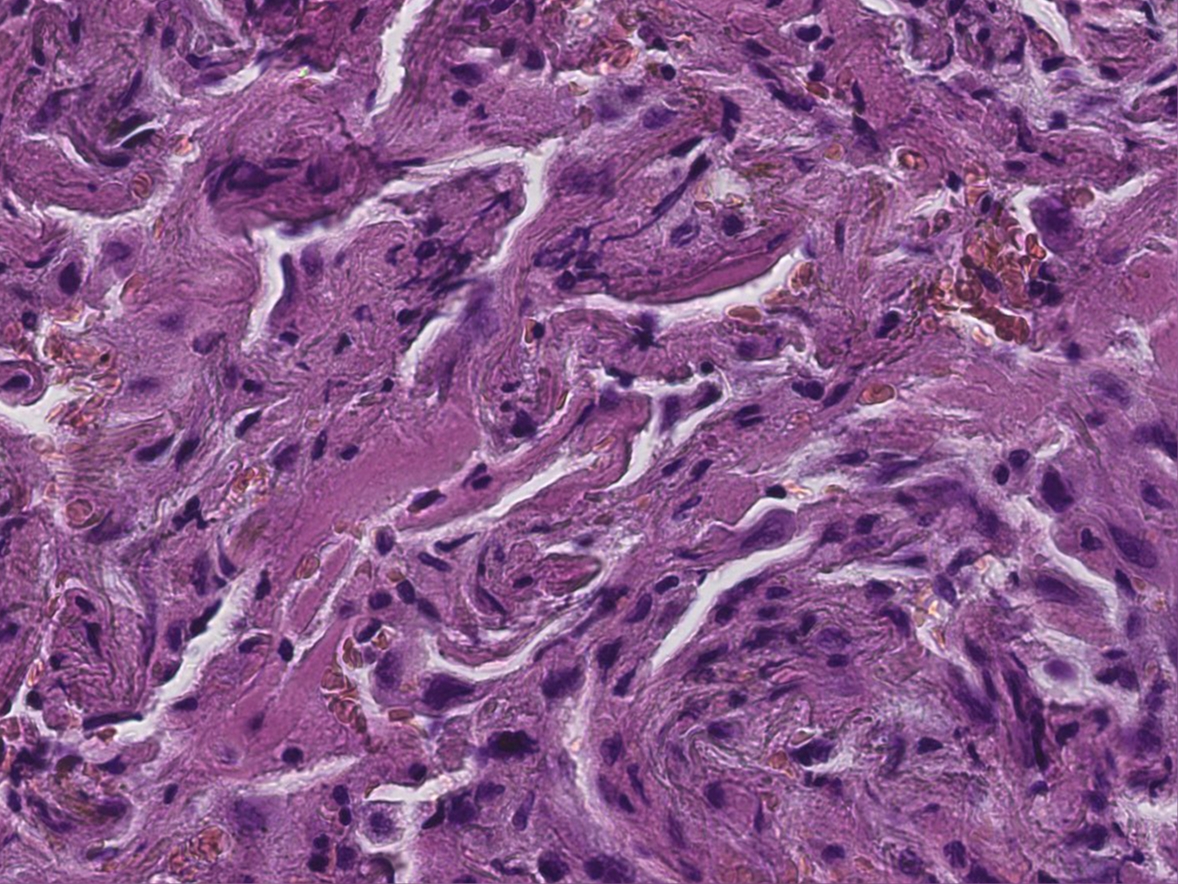
**

**Figure 4.** *Hyaline Membranes.* (x40 magnification)

Amorphous eosiniphilic materiel deposit on the alveolar wall.

*Case 2 pathological findings :*


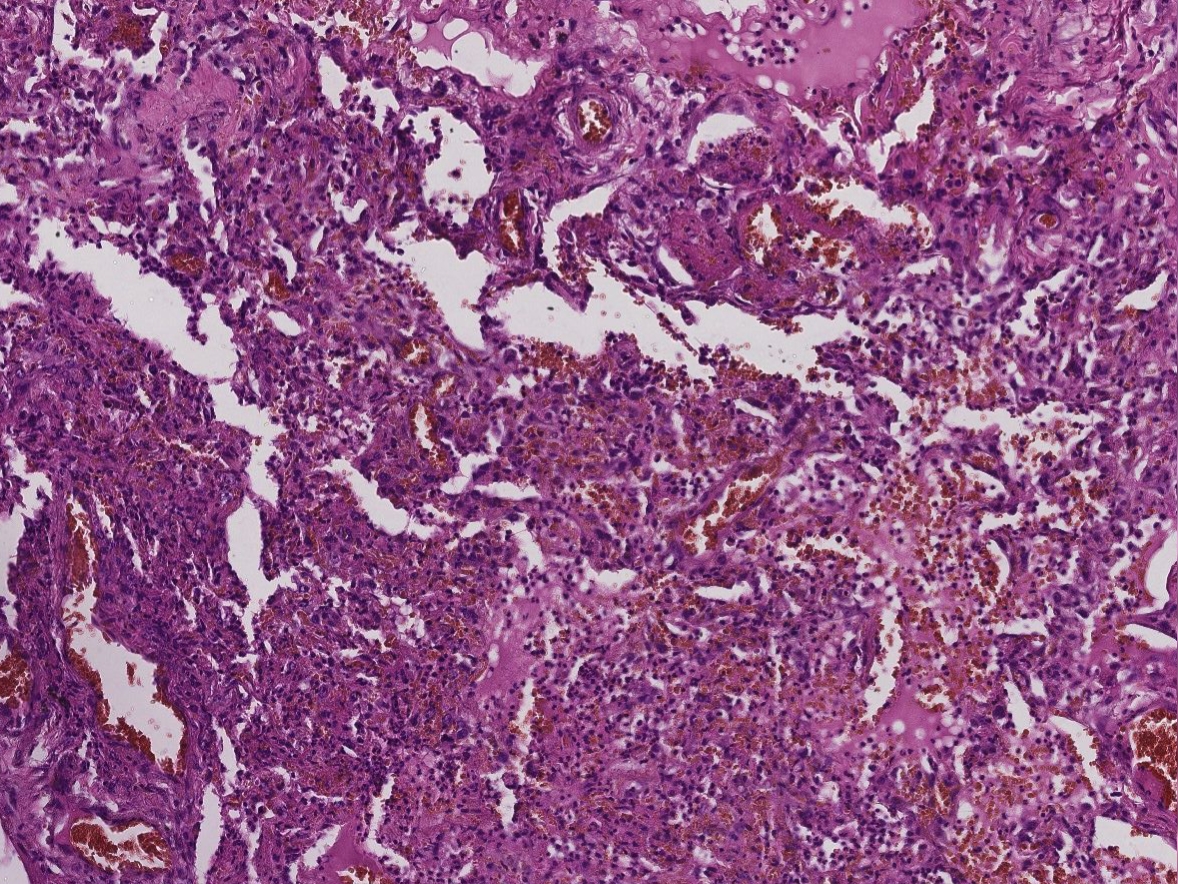


**Figure 5.** *Collapsed Alveoli demonstrating intense consolidation*. (x20 magnification)

The alveoli are filled with inflammatory exudates preventing the expansion of the alveolar sac.


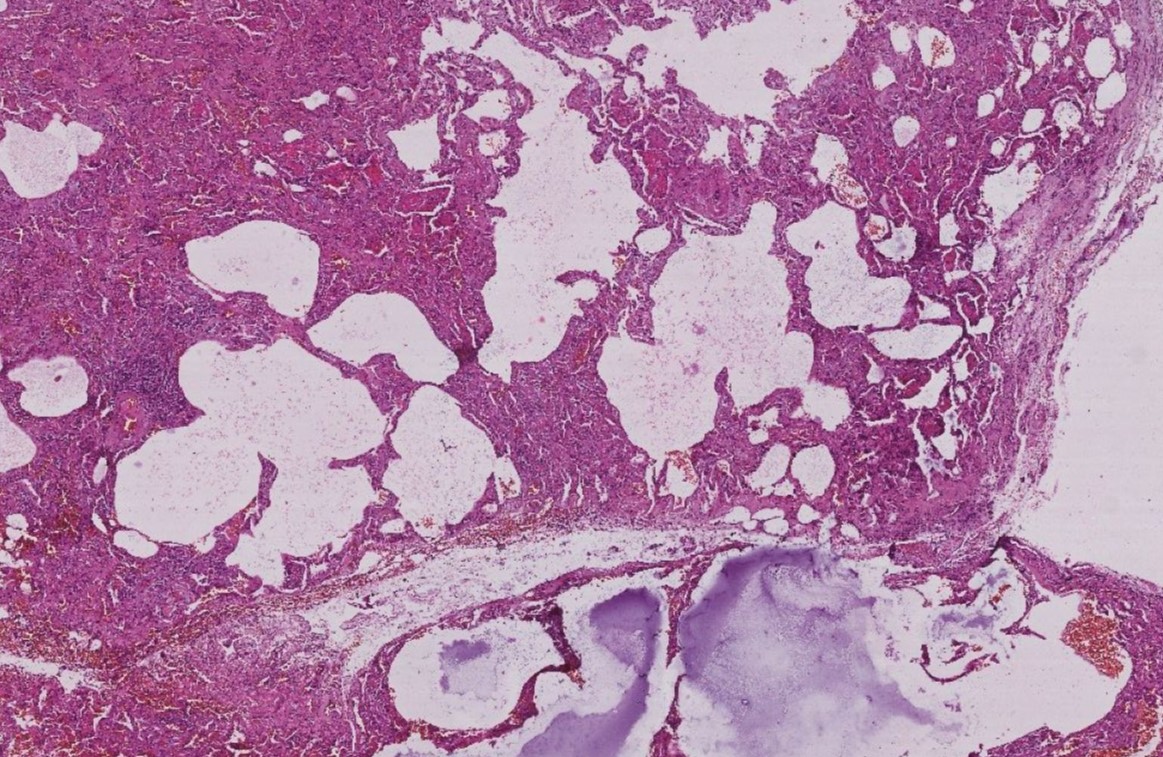


**Figure 6.** *Enlarged alveolar airspace.* (x20 magnification)

Enlargement of the alveolar spaces with thickening of the alveolar wall.


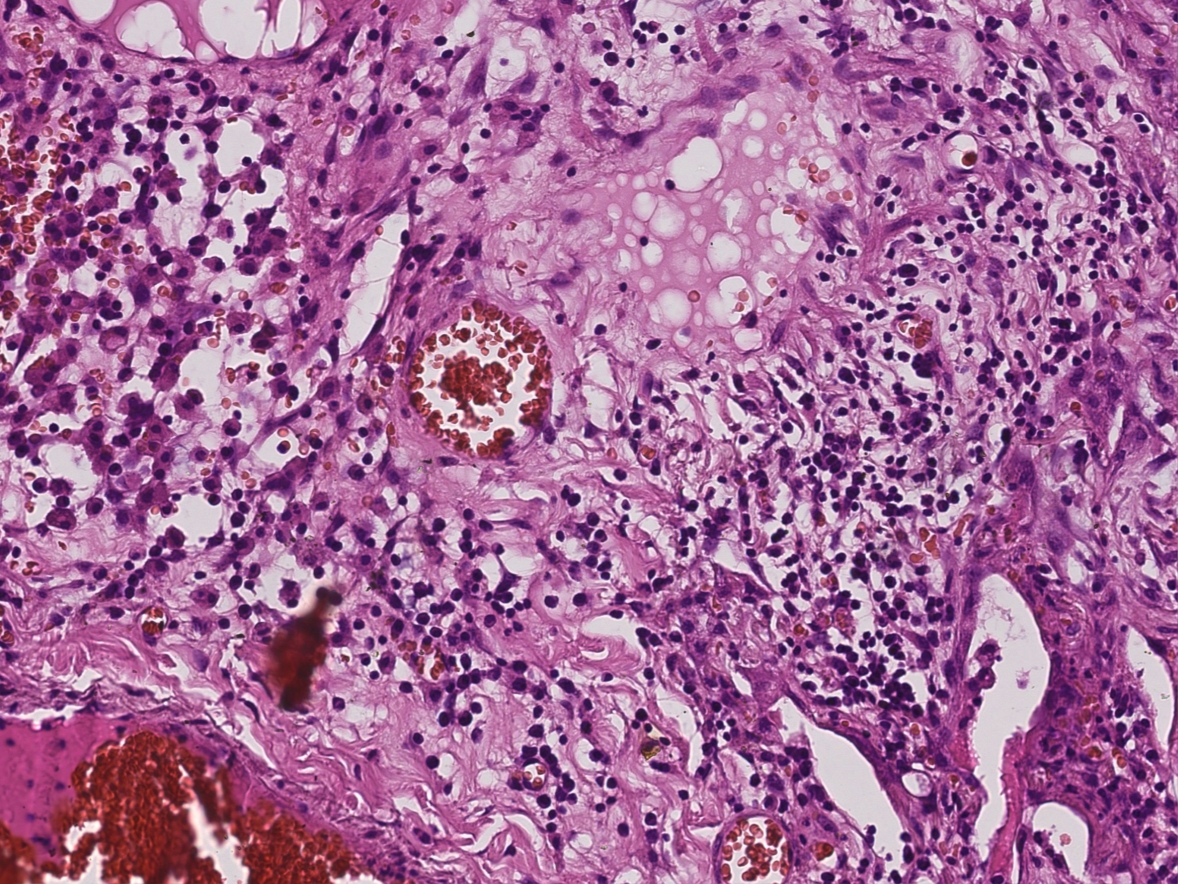


**Figure 7.** *Inflammatory infiltrate in the interstitial tissue*. (x 20 magnification)

Inflammatory cells (lymphocytes, plasmocytes, neutrophils) within the interstitial tissue.

*Case 3 pathological findings:*


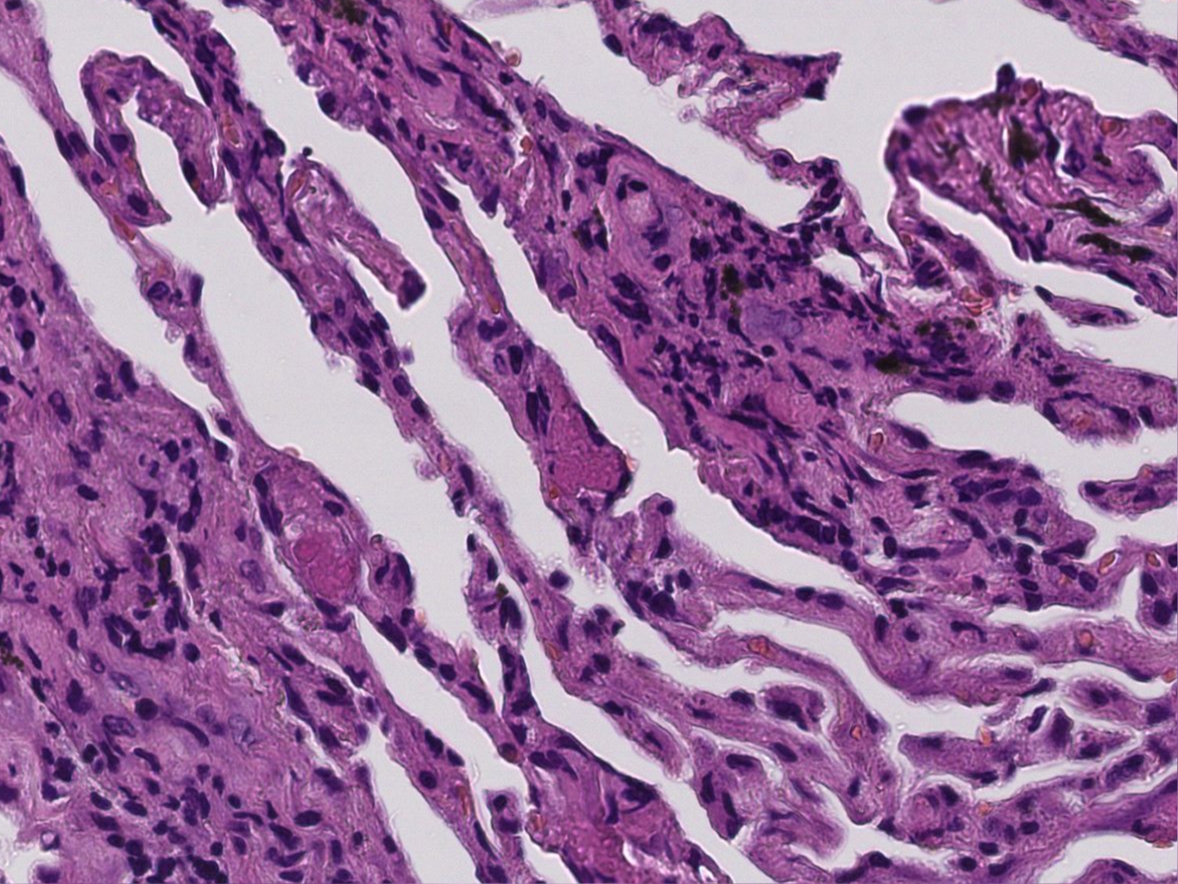


**Figure 8**. *Fibrin microthrombus.* (x40 magnification)

A small fibrin microthrombus in the alveolar capillaries.


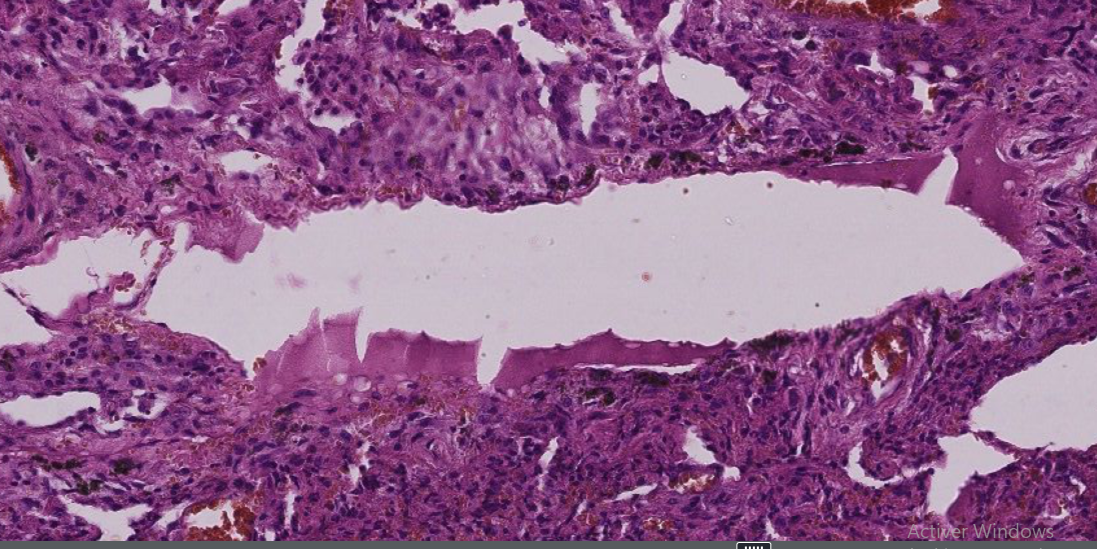


**Figure 9.** *Hyaline Membranes.* (x20 magnification)

Amorphous eosiniphilic materiel deposit on the alveolar wall.


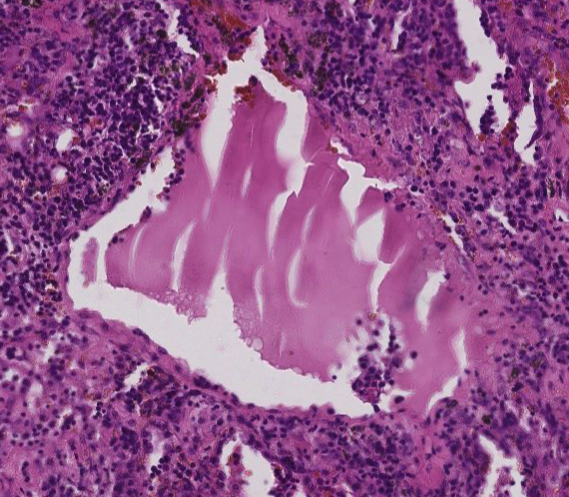


**Figure 10***. Protein exudates.* (x30 magnification)

Rich fluid in protein and cells leak through their alveolar walls.
